# Regulation of Latency and Reactivation by Human Cytomegalovirus miRNAs

**DOI:** 10.3390/pathogens10020200

**Published:** 2021-02-13

**Authors:** Nicole L. Diggins, Rebecca L. Skalsky, Meaghan H. Hancock

**Affiliations:** Vaccine and Gene Therapy Institute, Oregon Health and Science University, Beaverton, OR 97006, USA; diggins@ohsu.edu (N.L.D.); skalsky@ohsu.edu (R.L.S.)

**Keywords:** cytomegalovirus, miRNA, latency, signaling

## Abstract

Human cytomegalovirus (HCMV) encodes 22 mature microRNAs (miRNAs), which regulate a myriad of cellular processes, including vesicular trafficking, cell cycle progression, apoptosis, and immune evasion, as well as viral gene expression. Recent evidence points to a critical role for HCMV miRNAs in mediating latency in CD34^+^ hematopoietic progenitor cells through modulation of cellular signaling pathways, including attenuation of TGFβ and EGFR signaling. Moreover, HCMV miRNAs can act in concert with, or in opposition to, viral proteins in regulating host cell functions. Here, we comprehensively review the studies of HCMV miRNAs in the context of latency and highlight the novel processes that are manipulated by the virus using these small non-coding RNAs.

## 1. Introduction

Human cytomegalovirus (HCMV), a prototypic member of the beta-herpesvirus family, has a large, double-stranded DNA genome of approximately 230 kbp that encodes for greater than 170 proteins, as well as numerous long and small non-coding RNAs [1,2,3]. CMVs have co-evolved with their hosts over millions of years, and as such a careful balance between virus replication, viral latency, and host immune control has emerged. This intricate balance between virus and host results in a CMV seroprevalence of approximately 40–90% across different human populations [4]. While T-cell-mediated immunity helps keep viral replication in check in an immunocompetent host [5,6], immunocompromised individuals, such as those undergoing solid organ or hematopoietic stem cell transplants, are susceptible to CMV reactivation from latency and virus replication in numerous tissues and organs, causing significant morbidity and mortality [7,8].

CMVs can infect a remarkable breadth of cell types [9,10] and diverse transcriptional programs are elicited that represent distinct modes of infection. In many cell types, such as fibroblasts and smooth muscle cells, CMVs undergo lytic replication, whereby viral gene expression follows a strict program of immediate early (IE) followed by early (E) and then late (L) genes [11]. Subsequent virion assembly and egress results in the release of new virus particles that can go on to infect neighboring or infiltrating cells. A much more protracted replication cycle happens in endothelial and epithelial cells, which may be sources of long-term viral shedding and immune stimulation [12]. 

CMV latency, defined by maintenance of viral genomes in the absence of new virion production, is established when infected monocytes traffic to the bone marrow and seed infection of CD34^+^ hematopoietic progenitor cells (HPCs). The mechanisms by which the virus enters and exits latency have long been unclear, and the difficulty in enriching for rare latently infected cells in vivo has limited the study of natural latency. However, the development of in vitro systems that mimic aspects of the bone marrow stroma and maintain HPCs in their progenitor state has allowed for investigation into the role of specific gene products in latency and reactivation [13,14,15,16,17,18,19,20,21]. Transcriptional profiling during latent infection has suggested widespread, although very mild, gene expression from the latent genome [22,23,24]. Reactivation from latency occurs following encounters with external stimuli, such as proinflammatory cytokines, which alter the intracellular environment in a manner that favors efficient re-expression of IE, E, and L viral gene products [19,25,26,27]. The signaling pathways and viral and cellular gene products required for reactivation continue to be investigated and cellular differentiation along the myeloid lineage is necessary for navigating the reactivation process [27,28]. HCMV drives myeloid-specific differentiation, as well as trafficking of infected HPCs out of the bone marrow and their differentiation into tissue-specific macrophages through unknown mechanisms, which leads to the full cascade of viral gene expression and production of new infectious virions [5]. 

Over the past two decades, miRNAs have emerged as potent and cell-type-specific regulators of the host cell environment. These miRNAs are small, ~22 nucleotide non-coding RNAs that post-transcriptionally regulate gene expression [29]. Mature miRNAs are generated from hairpin secondary structures that arise from longer RNA polymerase II or polymerase III transcripts [30]. In the nucleus, primary (pri-) miRNAs are cleaved into precursor (pre-) miRNAs via the microprocessor complex, consisting of DGCR8 and the ribonuclease Drosha. Pre-miRNAs are then exported to the cytoplasm, whereupon a second cleavage event by the RNAse III endonuclease Dicer results in miRNA duplexes. The miRNA is subsequently loaded into an argonaute (Ago) protein, thereby releasing the passenger strand, and forming the minimal RNA-induced silencing complex (RISC). RISC utilizes the miRNA seed sequence region (nucleotides 2 through 8) to recognize and bind complementary regions of targeted transcripts. Association of RISC with a target RNA results in translational repression through inhibition of translation initiation or elongation factors or mRNA decay through recruitment of deadenylation factors [31,32]. Because of the short regions of complementarity utilized by miRNAs, they have the capacity to target up to hundreds of different genes [33], making them powerful regulators of gene expression. In fact, individual deletion of most major cellular miRNA families results in a wide array of defects in eukaryotic organisms [34,35,36].

miRNAs are encoded not only by eukaryotic organisms but also viruses, which utilize small RNAs to aid in their replication cycles [37]. Of the over 250 identified viral miRNAs to date, most are encoded by members of the herpesvirus family, including alpha, beta, and gamma herpesviruses. The first herpesvirus miRNAs were identified in cells latently infected with Epstein–Barr virus (EBV) [38] and subsequent functional studies have implicated the EBV miRNAs in latency maintenance [39,40,41]. Alpha and gamma herpesvirus miRNAs are found clustered in viral genomic regions that are known to be expressed during latency, suggesting roles in maintaining latent infection [42,43,44,45]. In contrast, CMV miRNAs are encoded throughout the viral genome rather than in discrete locations associated with CMV latent gene expression [46,47]. These observations would suggest roles for CMV miRNAs at multiple stages throughout infection; however, these discrepancies may also be due to our still-limited understanding of CMV gene expression during latent infection in vivo. 

In order to successfully establish a latent infection, HCMV must enter CD34^+^ HPCs, maintain its genome, and simultaneously support essential cellular functions to avoid cell death and detection by the innate and adaptive immune responses. Moreover, reactivation requires that the viral genome remains responsive only to appropriate reactivation cues while avoiding sub-optimal activation signals. Their non-immunogenic nature, along with the ability to target potentially hundreds of different transcripts, suggests that HCMV miRNAs could be key regulators of protein expression during latency, where other viral factors may not reach the expression threshold necessary to exert their functions. Notably, targets of HCMV miRNAs have been identified that play key roles in regulating CD34^+^ HPC proliferation and hematopoiesis, along with entry into and exit from latency.

## 2. HCMV miRNAs Expressed In Vitro and In Vivo

**Figure 1:**
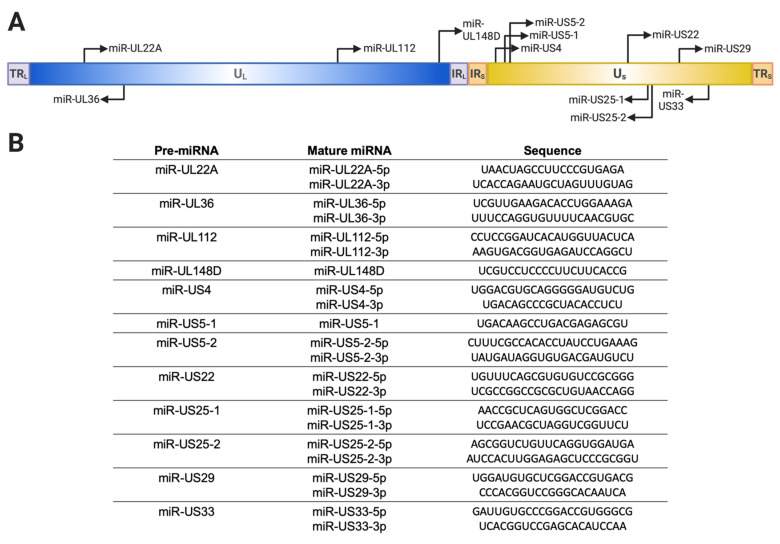
Schematic of microRNAs (miRNAs) encoded by Human cytomegalovirus (HCMV). (**A**) Locations and orientations of pre-miRNAs are shown as black arrows on the HCMV genome. TR_L/S_, tandem repeat long/short; U_L/S_, unique long/short; IR_L/S_, internal repeat long/short. (**B**) List of pre-miRNAs, associated mature miRNAs, and corresponding mature miRNA sequences are shown.

HCMV miRNAs were first described by Pfeffer et al. [48], who identified 9 pre-miRNA sequences in lytically infected cells; these were later independently confirmed by multiple groups [46,47,49]. Subsequently, next-generation sequencing analysis of lytically infected human fibroblasts revealed 22 mature HCMV miRNAs arising from a total of 12 pre-miRNAs (Figure 1) and further confirmed incorporation of the HCMV miRNAs into RISC [47]. miRNA expression has also been assessed in latently infected CD14^+^ monocytes and CD34^+^ HPCs using qRT-PCR [15,50,51,52]. These studies demonstrated that all HCMV miRNAs are expressed and abundantly detected at early times after infection of CD34^+^ HPCs (2–4 dpi). However, by 10–14 dpi, only a few miRNAs, such as miR-UL112-3p, miR-UL22A, and miR-UL148D, remain abundantly detectable. Given that only ~1 in 1000 to 1 in 10,000 cells contain viral genomes capable of reactivation [53], the expression pattern of HCMV miRNAs in truly latently infected cells awaits the ability to detect and enrich for these cells. 

HCMV miRNAs have been examined as potential biomarkers and as a means to predict CMV DNAemia in a number of disease settings, including during hematopoietic and solid organ transplantation. HCMV miRNAs have been detected in plasma and serum [54,55,56,57,58,59], whole blood [60], extracellular vesicles isolated from serum [61], saliva [62], and purified monocytes and PBMCs [52] in healthy and diseased individuals. The most commonly detected miRNAs, miR-US25-1, miR-UL112-3p, and miR-UL22A, are also amongst the most highly expressed during lytic and latent infection. HCMV miRNAs have also been detected in astrocytic tumors [63] and glioblastoma tissue [64].

## 3. HCMV miRNAs Involved in Latency Establishment and Maintenance

**Figure 2:**
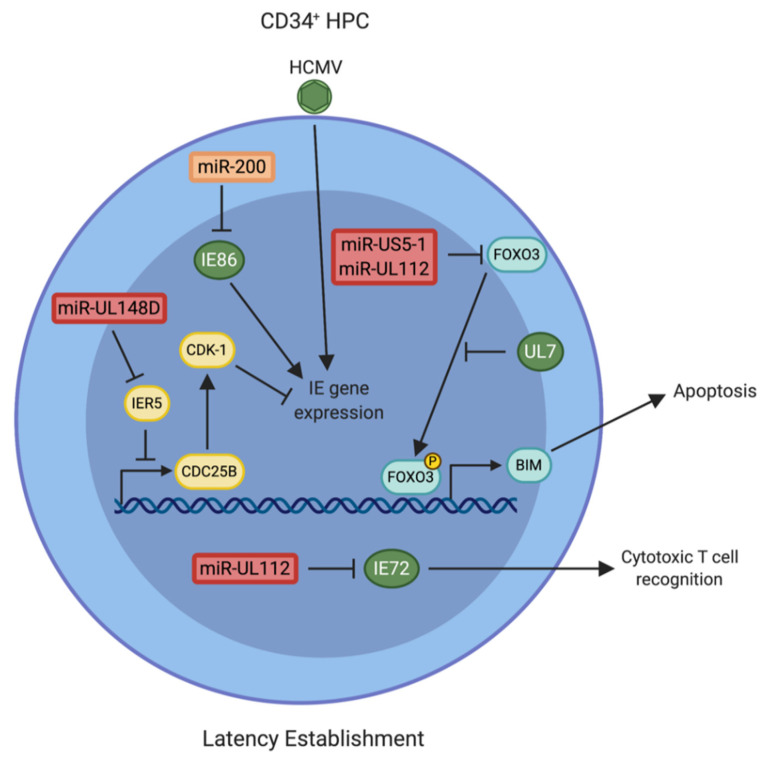
A model of Human cytomegalovirus (HCMV) microRNA (miRNA) regulation of latency establishment. Following viral entry into a CD34^+^ hematopoietic progenitor cell (HPC), HCMV immediate early (IE) gene expression must be silenced and host cell signaling must be remodeled to prevent apoptosis and immune recognition to promote latent HCMV infection. Furthermore, miR-UL112 directly targets HCMV immediate early gene IE72 to avoid recognition by cytotoxic T cells [67], while miR-UL148D targets Immediate early response 5 (IER5) to indirectly regulate IE gene expression through Cyclin-dependant Kinase-1 (CDK-1) [51]. In addition, a cellular miRNA family expressed in CD34^+^ HPCs targets HCMV IE86 to prevent lytic replication [84]. Additionally, miR-US5-1 and miR-UL112 act synergistically with HCMV US7 to downregulate Forkhead Box O3 (FOXO3a) and thereby prevent apoptosis during latency establishment [77]. HCMV proteins are shown in green and HCMV miRNAs are shown in red.

In order to establish latency, expression of the viral IE proteins, essential for launching the lytic cascade of viral gene expression, must be suppressed. HCMV miRNAs are involved in inhibiting IE gene expression through a variety of mechanisms. One of the first identified targets of HCMV miR-UL112-3p is the *UL123* transcript, encoding the major immediate early lytic protein IE72; pre-expression of miR-UL112-3p limits IE72 protein levels and limits viral DNA copy numbers in infected cells [65]. Further studies demonstrated that mutation of the miR-UL112-3p binding site within the *UL123* transcript alleviated miR-UL112-3p-mediated reduction of IE72 protein levels [66]. Interestingly, infection of CD14^+^ monocytes with the IE72 miR-UL112-3p binding site mutant revealed that miRNA targeting of IE72 is not needed for latency establishment or reactivation in vitro, but is important to limit IE gene expression and cytotoxic T cell recognition [67]. Thus, miR-UL112-3p targeting of IE72 may play an important role in maintaining the pool of latently infected cells within the host (Figure 2). Although not directly tested in the context of latent infection, miR-UL112-3p also targets HCMV UL112/UL113 and UL120/UL121 [65]. Moreover, other HCMV miRNAs target viral transcripts, potentially contributing to the establishment or maintenance of latent infection. Additionally, miR-US5-1 and miR-US5-2 target US7 [68], while miR-UL36 targets UL138 [69]. Of note, an miR-US5-2 homolog (miR-Rh183-1) encoded by Rhesus CMV (RhCMV) also targets the RhCMV US7 homolog (Rh186) [70]. The region encompassing this miRNA can be removed from the viral genome and the virus can still infect rhesus macaques and induce T cell responses to heterologous antigens [71], suggesting that reducing US7 expression through miRNA targeting is not essential for infection in vivo. 

In addition to directly regulating HCMV genes, HCMV miRNAs target a wide array of cellular transcripts, thereby altering the host cell environment during the establishment of latency. Pan et al. [51] showed that a miR-UL148D mutant virus was unable to establish latency in CD34^+^ HPCs and instead underwent a lytic infection cycle. The authors identified one direct target of miR-UL148D, IER5, which regulates the CDK-1 phosphatase CDC25B. During infection of CD34^+^ HPCs with a miR-UL148D mutant, levels of IER5 protein were significantly increased, while CDC25B showed a concordant decrease in expression. Studies have shown that CDC25B activates CDK-1 through dephosphorylation [72], which in turn inhibits transcription of HCMV *UL123* (IE72) [73]. Using the myeloid cell line Kasumi-3, the authors show that infection with the miR-UL148D mutant virus does not inhibit IE1 gene expression due to the enhanced phosphorylation of CDK-1 that occurs upon reduced CDC25B expression [51] (Figure 2). Thus, by interfering with expression of a cellular immediate early response gene, HCMV miR-UL148D indirectly regulates viral IE gene expression and latency establishment.

Latency establishment and maintenance require that the infected cells block host apoptotic responses. High-throughput analysis of HCMV miRNA targets in lytically infected human fibroblasts identified multiple targets related to apoptosis signaling, including FAS, FADD, CASP3, and CASP7 [74]. Additional studies performed in the absence of infection or using cell lines that are not permissive for HCMV latency have suggested that HCMV miRNAs can inhibit apoptosis through suppression of SLC25A6/ANT3 [75] and immediate early gene X-1 (IEX1) [76]. More recently, HCMV miR-US5-1 and miR-UL112-3p were shown to target FOXO3a [77], a member of the mammalian Forkhead Box O family of transcription factors that promotes mitochondrial-dependent and -independent mechanisms of apoptosis induction [78,79]. FOXO3a binds to the promoters of pro-apoptotic regulators such as Bcl-2-like protein 11 (Bim) and stimulates its expression [80]. The activity of FOXO3a is regulated by PI3K/AKT and MEK/ERK signaling, which mediate phosphorylation and translocation of FOXO3a to the cytoplasm [81,82,83]. The FOXO3a transcript is downregulated by HCMV miR-US5-1 and miR-UL112-3p and the protein is targeted for phosphorylation and inactivation by the HCMV FLT3L homolog UL7 [77]. Both the miRNAs and UL7 are expressed at early times post-infection of CD34^+^ HPC [15,77] and reduce FOXO3a levels and activity to limit the induction of apoptosis [77] in this cell type (Figure 2). This study demonstrates a coordination between an HCMV protein and HCMV miRNAs to promote survival of infected cells in a way that supports the establishment of HCMV latency. 

**Figure 3:**
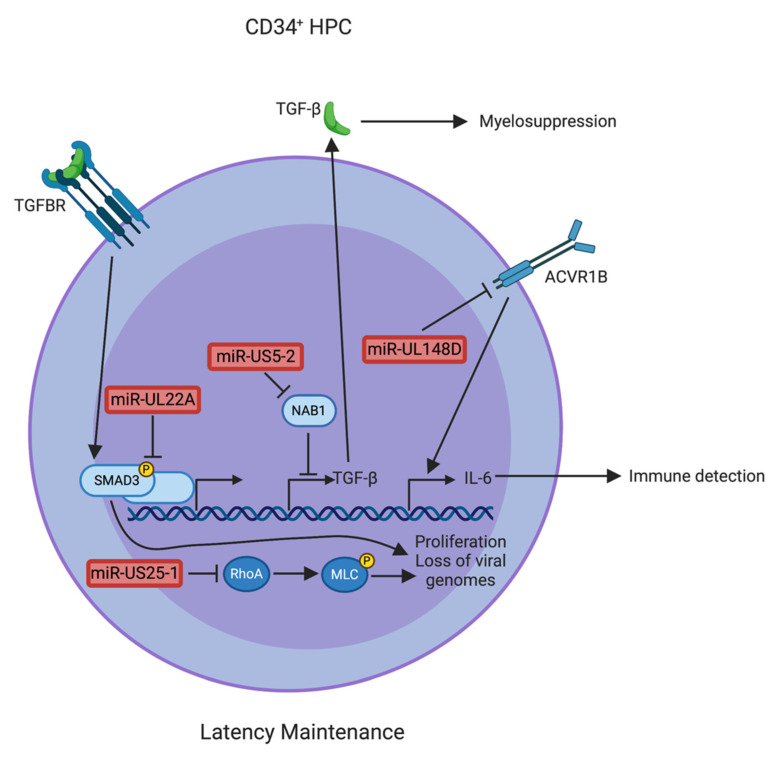
A model of Human cytomegalovirus (HCMV) factors that contribute to latency maintenance. Host cell signaling is modulated by HCMV proteins and microRNAs (miRNAs) to evade immune detection, limit proliferation, and suppress myeloid differentiation. Furthermore, miR-UL148D inhibits activin signaling to limit pro-inflammatory cytokine release from the latently infected cell [50]. Additionally, miR-US5-2 targets the transcriptional repressor NGFI-A-binding protein 1 (NAB1) to increase Transforming Growth Factor beta (TGFβ) production and secretion, resulting in myelosuppression. However, miR-UL22A blocks the TGFβ pathway to protect the infected cell from the effects of TGFβ and to maintain viral genomes during latency [16]. Moreover, miR-US25-1 also prevents the loss of viral genomes during latency by targeting the GTPase Ras homology family member A (RhoA) and inhibiting proliferation of latently infected CD34^+^ hematopoietic progenitor cells (HPCs) [88]. HCMV miRNAs are shown in red.

While limiting IE gene expression and preventing apoptosis are critical steps in latency establishment, very little is known about the viral and cellular factors required to maintain the latent genome in CD34^+^ HPCs. Recently, TGFβ signaling was identified as an important antiviral response during latency that affects viral genome maintenance [16]. HCMV miR-UL22A-5p and miR-UL22A-3p target SMAD3, a key transcription factor downstream of TGFβ binding to the TGFβ receptor (Figure 3). HCMV lacking the miR-UL22A hairpin does not block canonical TGFβ signaling in CD34^+^ HPCs and fails to reactivate from latency. Further examination determined that miR-UL22A mutant genomes were lost during latency, accounting for the lack of reactivation. A miR-UL22A mutant virus engineered to express an shRNA targeting SMAD3 from the miR-UL22A locus reverted the ΔmiR-UL22A phenotype to that of wild type (WT)—canonical TGFβ signaling was blocked in CD34^+^ HPCs and viral genomes were maintained and capable of reactivation [16]. These data indicate that targeting SMAD3 is an essential function of miR-UL22A during latent infection of CD34^+^ HPCs in order to maintain viral genomes capable of reactivation. Altogether, the studies described here emphasize the complexities surrounding HCMV miRNA regulation of both host and viral factors that contribute to the ability of HCMV to enter into and maintain latency in CD34^+^ HPCs. 

## 4. HCMV miRNAs Involved in Regulating CD34^+^ HPC Proliferation and Myelopoiesis

In order to regulate latency in CD34^+^ HPCs, the virus must participate in maintaining the quiescent state of the progenitor cell. Conversely, upon reactivation stimuli, the virus drives cell differentiation through the myeloid lineage. Additionally, because HCMV is not known to tether its genome to the host chromosomes during cell division, HCMV also actively limits the proliferation of the infected HPC. Thus, the virus carefully manipulates the homeostasis of infected CD34^+^ HPCs to aid in specific steps of its lifecycle, and evidence is emerging to suggest this occurs in part through the actions of viral miRNAs.

HCMV miR-US22 targets EGR-1 [15], which is an important modulator of CD34^+^ HPC proliferation [85]. EGR-1 is critical for promoting “stemness”—self renewal and a lack of differentiation—of CD34^+^ HPCs in the bone marrow niche in vivo [86,87]. Expression of an EGR-1 shRNA phenocopies the effect of miR-US22 in limiting proliferation of CD34^+^ HPCs [15]. Given that miR-US22 is not expressed during latent infection, this suggests that expression of miR-US22 either at the initial stages of infection or upon reactivation is important for limiting the proliferation of cells harboring viral genomes.

As an additional mechanism to limit cell proliferation during latent infection, HCMV miR-US25-1 targets RhoA, a GTPase critical for regulating actin dynamics [88]. miR-US25-1 directly targets the 3′ UTR of RhoA, thereby reducing protein expression, attenuating downstream signaling through myosin light chain II, and limiting the formation of the contractile ring required for cytokinesis (Figure 3). Notably, ΔmiR-US25-1-infected CD34^+^ HPCs proliferate significantly more than WT-infected cells, and reactivate from latency with a lower frequency compared to WT. Given that the ΔmiR-US25-1-infected cells proliferate more extensively, the lower frequency of reactivation is due to a lower proportion of genome-containing cells at the end of the latency culture [88]. Thus, miR-US25-1 targeting of RhoA uncovers a novel means of enriching for viral-genome-containing cells during latency. Along with RhoA, additional cell cycle regulators have been identified as targets of miR-US25-1 [89], suggesting that the miRNA may regulate proliferation during infection using multiple mechanisms.

HCMV-infected CD34^+^ HPCs not only show significant reduction in proliferation, but specific myeloid differentiation programs are also blocked by infection. For many years, it has been observed that HCMV infection is myelosuppressive, both in vitro and in vivo [16,90,91,92,93,94], but the mechanisms surrounding this myelosuppression were unknown. Recently, it was determined that latently infected CD34^+^ HPCs secrete the myelosuppressive cytokine TGFβ [16,95]. TGFβ expression is negatively regulated by the transcriptional repressor NAB1, which brings HDAC2 and other chromatin modifiers to the TGFβ promoter [96,97]. NAB1 is a target of HCMV miR-US5-2, and expression of either miR-US5-2 or a NAB1 siRNA induced the expression and secretion of TGFβ and limited myeloid colony formation in CD34^+^ HPCs [16] (Figure 3). In support of these findings, a miR-US5-2 mutant virus showed decreased TGFβ secretion and enhanced proliferation and myeloid colony formation compared to WT-infected CD34^+^ HPCs. Thus, through increased TGFβ production via downregulation of a transcriptional repressor, miR-US5-2 is capable of mediating myelosuppression in the local microenvironment during latent infection. Interestingly, HCMV also blocks canonical TGFβ signaling through targeting SMAD3 using the latently-expressed miRNAs miR-UL22A-5p and -3p (see above). Thus, while the virus stimulates TGFβ secretion, it protects itself from the consequences of TGFβ signaling within the infected cell (Figure 3). These data illustrate the incredible power that HCMV miRNAs can exert during latent infection to regulate both the intracellular and extracellular environment.

## 5. HCMV miRNAs Involved in Reactivation from Latency

**Figure 4:**
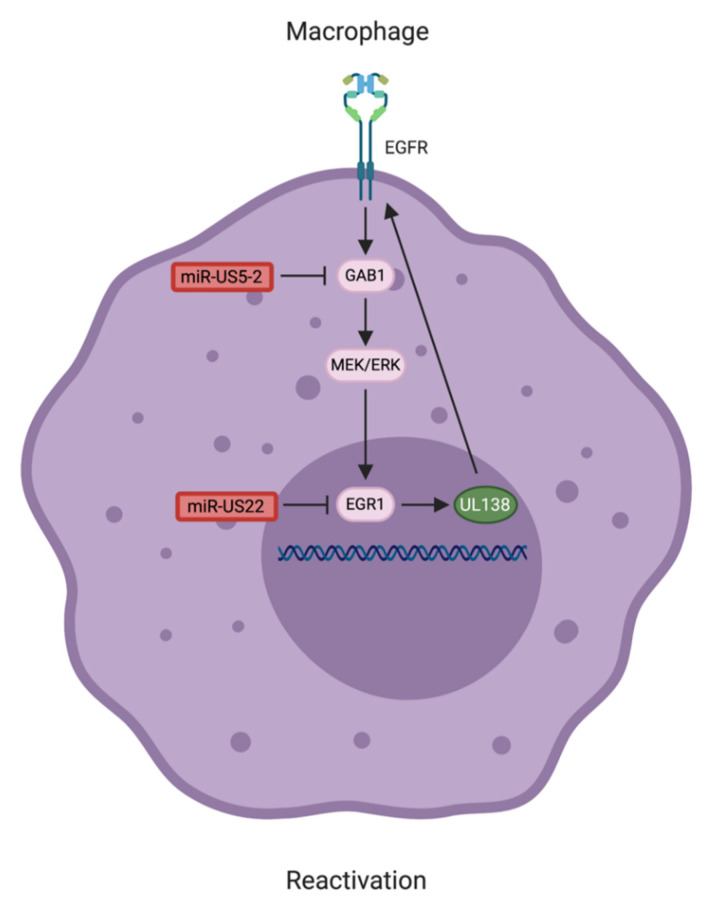
A model of Human cytomegalovirus (HCMV) microRNA- (miRNA)-mediated reactivation from latency. Reactivation from latency requires myeloid differentiation and attenuation of host cell signaling important for latency maintenance. HCMV miRNAs target multiple components of the Epidermal growth factor receptor (EGFR)/Mitogen activated protein kinase kinase (MEK)/Extracellular signal-regulated kinase (ERK) signaling pathway to interfere with *UL138* expression. HCMV UL138 promotes EGFR signaling, which is critical for latency maintenance [98]. EGFR signaling through Early growth response-1 (EGR-1), in turn, promotes expression of HCMV UL138, creating a feed-forward loop [14]. Although miR-US22 is not expressed during latency, it may instead act to block EGFR signaling during the early steps of reactivation [15]. Furthermore, miR-US5-2 also inhibits a component of the EGFR pathway and may contribute to HCMV reactivation [99]. HCMV proteins are shown in green and HCMV miRNAs are shown in red.

Reactivation from latency is a complex and multistep process that results in attenuation of host signaling pathways important for latency maintenance and the stimulation of other pathways involved in viral gene expression and cellular differentiation. Modelling HCMV reactivation from latency in CD34^+^ HPCs in vitro has proved technically difficult, and thus less is known about the role of HCMV miRNAs in this process. However, one HCMV miRNA implicated in the reactivation process is miR-US22, which targets the immediate early transcription factor EGR-1. Expression of miR-US22 reduces EGR-1 protein levels and blocks the EGFR/MEK/ERK-mediated stimulation of an EGR-1 transcriptional reporter [15]. Buehler et al. [14] determined that EGR-1 is involved in the expression of the latency-associated gene *UL138*. UL138 plays a role in maintaining the surface expression of EGFR and enhancing signaling through the MEK/ERK/EGR-1 pathway. Consequently, a feed-forward loop of EGFR signaling–*UL138* expression forms during HCMV latency that is critical for maintaining the latent state [14,98]. While miR-US22 is not detected during latent infection of CD34^+^ HPCs [15], it is thought to be re-expressed following reactivation when viral gene expression is re-initiated. The miR-US22-mediated downregulation of EGR-1 may contribute to breaking the EGFR-*UL138* signaling loop, and thus augmenting the viral reactivation process, although this remains to be directly tested. Additionally, miR-US5-2, through downregulating the EGFR adaptor protein GAB1, also indirectly regulates EGR-1 and UL138 expression [99]; however, the relevance of this interaction in latency and reactivation remains to be investigated. Collectively, these studies highlight a role for EGFR signaling as a critical switch between HCMV latency and reactivation [14,15,99]. Modulation of this signaling pathway at multiple stages of the viral life cycle is accomplished by an intricate interplay between HCMV proteins and miRNAs (Figure 4). 

## 6. HCMV miRNAs Involved in Evasion of Host Immune Responses

The most direct evidence of HCMV miRNA involvement in immune evasion during latency comes from the study of miR-UL148D and one of its targets, the activin receptor ACVR1B [50] (Figure 2). Using a monocyte infection model, Lau et al. [50] determined that ACVR1B levels were increased in cells infected with a ΔmiR-UL148D virus. While the lack of miR-UL148D expression had no effect on latency or reactivation in this model (in contrast to the work of Pan et al. [51], who demonstrated a lack of latency establishment in CD34^+^ HPCs infected with a ΔmiR-UL148D virus; see above), the authors demonstrated a significant upregulation of IL-6 secretion in response to activin A stimulation when miR-UL148D was deleted from the virus. Thus, miR-UL148D targeting of ACVR1B is involved in limiting proinflammatory cytokine levels during infection of monocytes.

Regulation of cytokine expression and release is a recurring theme among HCMV miRNAs. As mentioned above, miR-US5-2 targets NAB1, enhancing secretion of TGFβ during latent infection [16]. In lytic infection models, HCMV utilizes miR-US5-1 and miR-UL112-3p to limit the production of IL-6 and Regulated upon Activation, Normal T Cell Expressed and Presumably Secreted (RANTES) through regulation of IKKα and IKKβ expression and signaling through NFκB [100]. Additionally, miR-UL148D reduces production and secretion of RANTES by targeting the transcript directly [101]. As a means to limit secretion of TNFα and likely other cytokines, HCMV miRNAs significantly restructure the endocytic recycling compartment [102]. Although not all of these targets have been validated in latency models, these miRNAs could play a significant role in modulating the secretome of latently infected cells.

Finally, one of the earliest cellular targets identified for an HCMV miRNA is MHC class I-related chain B (MICB), a ligand for NK cells that triggers degranulation and killing of the interacting cell [103]. By targeting MICB for downregulation in infected cells, miR-UL112-3p partially inhibits NK-cell-mediated cytotoxicity in vitro [103]. Whether targeting MICB is important to protect latently infected cells from NK cell killing remains to be determined.

## 7. Cellular miRNAs Involved in Regulating Latency

While viral miRNAs target cellular and viral genes to regulate latency and reactivation in CD34^+^ HPCs, it is worth noting that specific cellular miRNAs are also involved in regulating HCMV IE gene expression. Members of the miR-200 family, including miR-200b, miR-200c, and miR-429, target the *UL122* (IE86) transcript (Figure 2). Mutation of the cellular miRNA binding site in the *UL122* transcript resulted in increased IE86 protein expression and enhanced lytic replication in CD34^+^ HPCs [84]. The miR-200 family members are more highly expressed in less-differentiated cells; thus, these data suggest that HCMV may have evolved to utilize cellular miRNAs to contribute to the regulation of IE expression in specific cell types. How the virus overcomes this repression during viral reactivation remains to be determined. HCMV latent infection downregulates expression of miR-92a, resulting in increased GATA-2 and IL-10 expression, which enhances viral DNA content in infected cells [104]. Additionally, miR-UL112-3p was found to act cooperatively with the cellular miRNA miR -376a for optimal downregulation of MICB during infection [105]. Finally, HCMV encodes a region between UL144 and UL145 that binds cellular miR-17 and miR-20a and reduces their levels during lytic infection. Termed the miRNA decay element (miRDE), the function of this region is not completely understood but may play a role in cell cycle regulation [106]. Since miR-17 family members play important roles in CD34^+^ HPC biology [107], it is possible that the miRDE region has important functions during HCMV latency that remain to be discovered.

## 8. Conclusions and Future Perspectives

In comparison to other herpesviruses, the species specificity and challenges inherent in HCMV latency model systems has hampered the understanding of HCMV miRNA targets during latency. Despite these challenges, the role of HCMV miRNAs in manipulating CD34^+^ HPC proliferation and myelopoiesis, as well as entry into and exit from latency, are beginning to be appreciated (Table 1). While originally considered fine-tuners of protein expression, study of HCMV miRNAs has uncovered a significant impact for these viral small non-coding RNAs on the host cell and its microenvironment. Emerging evidence points to a complex interplay between the viral proteins and miRNAs expressed during latent infection in regulating signaling pathways to establish (Figure 2), maintain (Figure 3), and reactivate from latency (Figure 4). It is inevitable that a more complete understanding of the HCMV miRNA targetome during latent infection will uncover further novel and exploitable means of viral miRNA regulation of host gene expression, providing greater insight into how HCMV regulates CD34^+^ HPC biology.

## Figures and Tables

**Table 1:** Roles of Human cytomegalovirus (HCMV) microRNAs (miRNAs) in latency and reactivation. List of HCMV-encoded miRNAs are shown along with their roles in latency and reactivation, or other roles not yet tested in cells that support latent infection.

miRNA	Role in Latency/Reactivation	Other Roles
**miR-US4-5p**	Unknown	
**miR-US4-3p**	Unknown	Targets CASP7, may affect apoptosis signaling [74]
**miR-US5-1**	Inhibits apoptosis during latency establishment via FOXO3a downregulation [77]	Targets HCMV US7 [68]
		Inhibits production of IL-6 and RANTES through IKKα and IKKβ targeting [100]
		Restructures the endocytic recycling compartment to limit cytokine secretion [102]
		Targets FADD, may affect apoptosis signaling [74]
**miR-US5-2**	Promotes TGFβ secretion by targeting the transcriptional repressor NAB1, mediates myelosuppression [16]	Targets HCMV US7 [68]
		Targets GAB1 to indirectly regulation EGR-1 and HCMV UL138 expression [99]
		Restructures the endocytic recycling compartment to limit cytokine secretion [102]
		Targets FAS, may affect apoptosis signaling [74]
**miR-UL22A-5p**	Targets SMAD3 to limit TGFβ signaling and maintain viral genomes during latency [16]	
**miR-UL22A-3p**	Targets SMAD3 to limit TGFβ signaling and maintain viral genomes during latency [16]	Targets CASP7, may affect apoptosis signaling [74]
**miR-US22**	Disrupts EGFR-UL138 signaling loop by targeting EGR-1 to induce viral reactivation [14]	
	Inhibits CD34^+^ HPC proliferation by targeting EGR-1 [15]	
**miR-US25-1**	Targets RhoA to limit proliferation and maintain viral genomes during latency [88]	
**miR-US25-2-5p**	Unknown	
**miR-US25-2-3p**	Unknown	Targets CASP3, may affect apoptosis signaling [74]
**miR-US29**	Unknown	
**miR-US33**	Unknown	
**miR-UL36**	Unknown	Targets HCMV UL138 [69]
**miR-UL36-5p**	Unknown	Targets SCL25A6/ANT3, inhibits apoptosis [75]
**miR-UL36-3p**	Unknown	Targets FAS, may affect apoptosis signaling [74]
**miR-UL112-3p**	Inhibits apoptosis during latency establishment via FOXO3a downregulation [77]	Inhibits production of IL-6 and RANTES through IKKα and IKKβ targeting [100]
	Targets HCMV IE72 to limit IE gene expression and CTL recognition [67]	Targets HCMV UL112/113 and UL120/121 [65]
		Limits NK cell-mediated cytotoxicity through MICB targeting [103]
		Restructures the endocytic recycling compartment to limit cytokine secretion [102]
**miR-UL148D**	Promotes latency establishment by indirectly inhibiting IE gene expression via IER5 downregulation [51]	Targets RANTES [101]
	Targets ACVR1B to limit proinflammatory cytokine levels [50]	Targets IEX1, inhibits apoptosis [76]

## Data Availability

Data sharing not applicable.

## References

[1] Murphy E., Yu D., Grimwood J., Schmutz J., Dickson M., Jarvis M.A., Hahn G., Nelson J.A., Myers R.M., Shenk T.E. (2003). Coding potential of laboratory and clinical strains of human cytomegalovirus. Proc. Natl. Acad. Sci. USA.

[2] Gatherer D., Seirafian S., Cunningham C., Holton M., Dargan D.J., Baluchova K., Hector R.D., Galbraith J., Herzyk P., Wilkinson G.W. (2011). High-resolution human cytomegalovirus transcriptome. Proc. Natl. Acad. Sci. USA.

[3] Stern-Ginossar N., Weisburd B., Michalski A., Le V.T., Hein M.Y., Huang S.X., Ma M., Shen B., Qian S.B., Hengel H. (2012). Decoding human cytomegalovirus. Science.

[4] Manicklal S., Emery V.C., Lazzarotto T., Boppana S.B., Gupta R.K. (2013). The “silent” global burden of congenital cytomegalovirus. Clin. Microbiol. Rev..

[5] Goodrum F. (2016). Human Cytomegalovirus Latency: Approaching the Gordian Knot. Annu. Rev. Virol..

[6] Griffiths P., Baraniak I., Reeves M. (2015). The pathogenesis of human cytomegalovirus. J. Pathol..

[7] Ljungman P., Hakki M., Boeckh M. (2011). Cytomegalovirus in hematopoietic stem cell transplant recipients. Hematol. Oncol. Clin. N. Am..

[8] Ramanan P., Razonable R.R. (2013). Cytomegalovirus infections in solid organ transplantation: A review. Infect. Chemother..

[9] Li G., Kamil J.P. (2016). Viral Regulation of Cell Tropism in Human Cytomegalovirus. J. Virol..

[10] Sinzger C., Hahn G., Digel M., Katona R., Sampaio K.L., Messerle M., Hengel H., Koszinowski U., Brune W., Adler B. (2008). Cloning and sequencing of a highly productive, endotheliotropic virus strain derived from human cytomegalovirus TB40/E. J. Gen. Virol..

[11] Wathen M.W., Stinski M.F. (1982). Temporal patterns of human cytomegalovirus transcription: Mapping the viral RNAs synthesized at immediate early, early, and late times after infection. J. Virol..

[12] Gerna G., Kabanova A., Lilleri D. (2019). Human Cytomegalovirus Cell Tropism and Host Cell Receptors. Vaccines (Basel).

[13] Goodrum F.D., Jordan C.T., High K., Shenk T. (2002). Human cytomegalovirus gene expression during infection of primary hematopoietic progenitor cells: A model for latency. Proc. Natl. Acad. Sci. USA.

[14] Buehler J., Carpenter E., Zeltzer S., Igarashi S., Rak M., Mikell I., Nelson J.A., Goodrum F. (2019). Host signaling and EGR1 transcriptional control of human cytomegalovirus replication and latency. PLoS Pathog..

[15] Mikell I., Crawford L.B., Hancock M.H., Mitchell J., Buehler J., Goodrum F., Nelson J.A. (2019). HCMV miR-US22 down-regulation of EGR-1 regulates CD34+ hematopoietic progenitor cell proliferation and viral reactivation. PLoS Pathog..

[16] Hancock M.H., Crawford L.B., Pham A.H., Mitchell J., Struthers H.M., Yurochko A.D., Caposio P., Nelson J.A. (2020). Human Cytomegalovirus miRNAs Regulate TGF-beta to Mediate Myelosuppression while Maintaining Viral Latency in CD34(+) Hematopoietic Progenitor Cells. Cell Host Microbe.

[17] Crawford L.B., Kim J.H., Collins-McMillen D., Lee B.J., Landais I., Held C., Nelson J.A., Yurochko A.D., Caposio P. (2018). Human Cytomegalovirus Encodes a Novel FLT3 Receptor Ligand Necessary for Hematopoietic Cell Differentiation and Viral Reactivation. mBio.

[18] Crawford L.B., Caposio P., Kreklywich C., Pham A.H., Hancock M.H., Jones T.A., Smith P.P., Yurochko A.D., Nelson J.A., Streblow D.N. (2019). Human Cytomegalovirus US28 Ligand Binding Activity Is Required for Latency in CD34(+) Hematopoietic Progenitor Cells and Humanized NSG Mice. mBio.

[19] Umashankar M., Goodrum F. (2014). Hematopoietic long-term culture (hLTC) for human cytomegalovirus latency and reactivation. Methods Mol. Biol..

[20] Krishna B.A., Wass A.B., Dooley A.L., O’Connor C.M. (2021). CMV-encoded GPCR pUL33 activates CREB and facilitates its recruitment to the MIE locus for efficient viral reactivation. J. Cell Sci..

[21] Krishna B.A., Wass A.B., O’Connor C.M. (2020). Activator protein-1 transactivation of the major immediate early locus is a determinant of cytomegalovirus reactivation from latency. Proc. Natl. Acad. Sci. USA.

[22] Rossetto C.C., Tarrant-Elorza M., Pari G.S. (2013). Cis and trans acting factors involved in human cytomegalovirus experimental and natural latent infection of CD14 (+) monocytes and CD34 (+) cells. PLoS Pathog..

[23] Cheng S., Caviness K., Buehler J., Smithey M., Nikolich-Zugich J., Goodrum F. (2017). Transcriptome-wide characterization of human cytomegalovirus in natural infection and experimental latency. Proc. Natl. Acad. Sci. USA.

[24] Shnayder M., Nachshon A., Krishna B., Poole E., Boshkov A., Binyamin A., Maza I., Sinclair J., Schwartz M., Stern-Ginossar N. (2018). Defining the Transcriptional Landscape during Cytomegalovirus Latency with Single-Cell RNA Sequencing. mBio.

[25] Hakki M., Goldman D.C., Streblow D.N., Hamlin K.L., Krekylwich C.N., Fleming W.H., Nelson J.A. (2014). HCMV infection of humanized mice after transplantation of G-CSF-mobilized peripheral blood stem cells from HCMV-seropositive donors. Biol. Blood Marrow Transplant..

[26] Crawford L.B., Streblow D.N., Hakki M., Nelson J.A., Caposio P. (2015). Humanized mouse models of human cytomegalovirus infection. Curr. Opin. Virol..

[27] Smith M.S., Goldman D.C., Bailey A.S., Pfaffle D.L., Kreklywich C.N., Spencer D.B., Othieno F.A., Streblow D.N., Garcia J.V., Fleming W.H. (2010). Granulocyte-colony stimulating factor reactivates human cytomegalovirus in a latently infected humanized mouse model. Cell Host Microbe.

[28] Soderberg-Naucler C., Fish K.N., Nelson J.A. (1997). Reactivation of latent human cytomegalovirus by allogeneic stimulation of blood cells from healthy donors. Cell.

[29] Gebert L.F.R., MacRae I.J. (2019). Regulation of microRNA function in animals. Nat. Rev. Mol. Cell Biol..

[30] Ha M., Kim V.N. (2014). Regulation of microRNA biogenesis. Nat. Rev. Mol. Cell Biol..

[31] Filipowicz W., Bhattacharyya S.N., Sonenberg N. (2008). Mechanisms of post-transcriptional regulation by microRNAs: Are the answers in sight?. Nat. Rev. Genet..

[32] Cai Y., Yu X., Hu S., Yu J. (2009). A brief review on the mechanisms of miRNA regulation. Genom. Proteom. Bioinform..

[33] Bartel D.P. (2009). MicroRNAs: Target recognition and regulatory functions. Cell.

[34] Alvarez-Saavedra E., Horvitz H.R. (2010). Many families of C. elegans microRNAs are not essential for development or viability. Curr. Biol..

[35] Shaw W.R., Armisen J., Lehrbach N.J., Miska E.A. (2010). The conserved miR-51 microRNA family is redundantly required for embryonic development and pharynx attachment in Caenorhabditis elegans. Genetics.

[36] Bartel D.P. (2018). Metazoan MicroRNAs. Cell.

[37] Kincaid R.P., Sullivan C.S. (2012). Virus-encoded microRNAs: An overview and a look to the future. PLoS Pathog..

[38] Pfeffer S., Zavolan M., Grasser F.A., Chien M., Russo J.J., Ju J., John B., Enright A.J., Marks D., Sander C. (2004). Identification of virus-encoded microRNAs. Science.

[39] Gregorovic G., Boulden E.A., Bosshard R., Elgueta Karstegl C., Skalsky R., Cullen B.R., Gujer C., Ramer P., Munz C., Farrell P.J. (2015). Epstein-Barr Viruses (EBVs) Deficient in EBV-Encoded RNAs Have Higher Levels of Latent Membrane Protein 2 RNA Expression in Lymphoblastoid Cell Lines and Efficiently Establish Persistent Infections in Humanized Mice. J. Virol..

[40] Albanese M., Tagawa T., Buschle A., Hammerschmidt W. (2017). MicroRNAs of Epstein-Barr Virus Control Innate and Adaptive Antiviral Immunity. J. Virol..

[41] Munz C. (2019). Latency and lytic replication in Epstein-Barr virus-associated oncogenesis. Nat. Rev. Microbiol..

[42] Umbach J.L., Kramer M.F., Jurak I., Karnowski H.W., Coen D.M., Cullen B.R. (2008). MicroRNAs expressed by herpes simplex virus 1 during latent infection regulate viral mRNAs. Nature.

[43] Tang S., Patel A., Krause P.R. (2009). Novel less-abundant viral microRNAs encoded by herpes simplex virus 2 latency-associated transcript and their roles in regulating ICP34.5 and ICP0 mRNAs. J. Virol..

[44] Cai X., Lu S., Zhang Z., Gonzalez C.M., Damania B., Cullen B.R. (2005). Kaposi’s sarcoma-associated herpesvirus expresses an array of viral microRNAs in latently infected cells. Proc. Natl. Acad. Sci. USA.

[45] Samols M.A., Hu J., Skalsky R.L., Renne R. (2005). Cloning and identification of a microRNA cluster within the latency-associated region of Kaposi’s sarcoma-associated herpesvirus. J. Virol..

[46] Grey F., Antoniewicz A., Allen E., Saugstad J., McShea A., Carrington J.C., Nelson J. (2005). Identification and characterization of human cytomegalovirus-encoded microRNAs. J. Virol..

[47] Stark T.J., Arnold J.D., Spector D.H., Yeo G.W. (2012). High-resolution profiling and analysis of viral and host small RNAs during human cytomegalovirus infection. J. Virol..

[48] Pfeffer S., Sewer A., Lagos-Quintana M., Sheridan R., Sander C., Grasser F.A., van Dyk L.F., Ho C.K., Shuman S., Chien M. (2005). Identification of microRNAs of the herpesvirus family. Nat. Methods.

[49] Dunn W., Trang P., Zhong Q., Yang E., van Belle C., Liu F. (2005). Human cytomegalovirus expresses novel microRNAs during productive viral infection. Cell. Microbiol..

[50] Lau B., Poole E., Krishna B., Sellart I., Wills M.R., Murphy E., Sinclair J. (2016). The Expression of Human Cytomegalovirus MicroRNAn miR-UL148D during Latent Infection in Primary Myeloid Cells Inhibits Activin A-triggered Secretion of IL-6. Sci. Rep..

[51] Pan C., Zhu D., Wang Y., Li L., Li D., Liu F., Zhang C.Y., Zen K. (2016). Human Cytomegalovirus miR-UL148D Facilitates Latent Viral Infection by Targeting Host Cell Immediate Early Response Gene 5. PLoS Pathog..

[52] Meshesha M.K., Bentwich Z., Solomon S.A., Avni Y.S. (2016). In vivo expression of human cytomegalovirus (HCMV) microRNAs during latency. Gene.

[53] Larsson S., Soderberg-Naucler C., Wang F.Z., Moller E. (1998). Cytomegalovirus DNA can be detected in peripheral blood mononuclear cells from all seropositive and most seronegative healthy blood donors over time. Transfusion.

[54] Ding M., Wang X., Wang C., Liu X., Zen K., Wang W., Zhang C.Y., Zhang C. (2017). Distinct expression profile of HCMV encoded miRNAs in plasma from oral lichen planus patients. J. Transl. Med..

[55] Li S., Zhu J., Zhang W., Chen Y., Zhang K., Popescu L.M., Ma X., Lau W.B., Rong R., Yu X. (2011). Signature microRNA expression profile of essential hypertension and its novel link to human cytomegalovirus infection. Circulation.

[56] Mohammad A.A., Rahbar A., Lui W.O., Davoudi B., Catrina A., Stragliotto G., Mellbin L., Hamsten A., Ryden L., Yaiw K.C. (2014). Detection of circulating hcmv-miR-UL112-3p in patients with glioblastoma, rheumatoid arthritis, diabetes mellitus and healthy controls. PLoS ONE.

[57] Zhou W., Wang C., Ding M., Bian Y., Zhong Y., Shen H., Wang J., Zhang C.Y., Zhang C. (2020). Different expression pattern of human cytomegalovirus-encoded microRNAs in circulation from virus latency to reactivation. J. Transl. Med..

[58] Talaya A., Gimenez E., Pascual M.J., Gago B., Pinana J.L., Hernandez-Boluda J.C., Vazquez L., Garcia M., Serrano D., Hernandez M. (2020). An investigation of the utility of plasma Cytomegalovirus (CMV) microRNA detection to predict CMV DNAemia in allogeneic hematopoietic stem cell transplant recipients. Med. Microbiol. Immunol..

[59] Kawano Y., Iwata S., Kawada J., Gotoh K., Suzuki M., Torii Y., Kojima S., Kimura H., Ito Y. (2013). Plasma viral microRNA profiles reveal potential biomarkers for chronic active Epstein-Barr virus infection. J. Infect. Dis..

[60] Lisboa L.F., Egli A., O’Shea D., Asberg A., Hartmann A., Rollag H., Pang X.L., Tyrrell D.L., Kumar D., Humar A. (2015). Hcmv-miR-UL22A-5p: A Biomarker in Transplantation With Broad Impact on Host Gene Expression and Potential Immunological Implications. Am. J. Transplant..

[61] Zhang J., Huang Y., Wang Q., Ma Y., Qi Y., Liu Z., Deng J., Ruan Q. (2020). Levels of human cytomegalovirus miR-US25-1-5p and miR-UL112-3p in serum extracellular vesicles from infants with HCMV active infection are significantly correlated with liver damage. Eur. J. Clin. Microbiol. Infect. Dis..

[62] Waters S., Lee S., Munyard K., Irish A., Price P., Wang B.H. (2020). Human Cytomegalovirus-Encoded microRNAs Can Be Found in Saliva Samples from Renal Transplant Recipients. Noncoding RNA.

[63] Deshpande R.P., Panigrahi M., Chandrasekhar Y.B.V.K., Babu P.P. (2018). Profiling of microRNAs modulating cytomegalovirus infection in astrocytoma patients. Neurol. Sci..

[64] Liang Q., Wang K., Wang B., Cai Q. (2017). HCMV-encoded miR-UL112-3p promotes glioblastoma progression via tumour suppressor candidate 3. Sci. Rep..

[65] Grey F., Meyers H., White E.A., Spector D.H., Nelson J. (2007). A human cytomegalovirus-encoded microRNA regulates expression of multiple viral genes involved in replication. PLoS Pathog..

[66] Murphy E., Vanicek J., Robins H., Shenk T., Levine A.J. (2008). Suppression of immediate-early viral gene expression by herpesvirus-coded microRNAs: Implications for latency. Proc. Natl. Acad. Sci. USA.

[67] Lau B., Poole E., Van Damme E., Bunkens L., Sowash M., King H., Murphy E., Wills M., Van Loock M., Sinclair J. (2016). Human cytomegalovirus miR-UL112-1 promotes the down-regulation of viral immediate early-gene expression during latency to prevent T-cell recognition of latently infected cells. J. Gen. Virol..

[68] Tirabassi R., Hook L., Landais I., Grey F., Meyers H., Hewitt H., Nelson J. (2011). Human cytomegalovirus US7 is regulated synergistically by two virally encoded microRNAs and by two distinct mechanisms. J. Virol.

[69] Huang Y., Qi Y., Ma Y., He R., Ji Y., Sun Z., Ruan Q. (2013). Down-regulation of human cytomegalovirus UL138, a novel latency-associated determinant, by hcmv-miR-UL36. J. Biosci..

[70] Hancock M.H., Tirabassi R.S., Nelson J.A. (2012). Rhesus cytomegalovirus encodes seventeen microRNAs that are differentially expressed in vitro and in vivo. Virology.

[71] Hansen S.G., Sacha J.B., Hughes C.M., Ford J.C., Burwitz B.J., Scholz I., Gilbride R.M., Lewis M.S., Gilliam A.N., Ventura A.B. (2013). Cytomegalovirus vectors violate CD8+ T cell epitope recognition paradigms. Science.

[72] Lindqvist A., Kallstrom H., Lundgren A., Barsoum E., Rosenthal C.K. (2005). Cdc25B cooperates with Cdc25A to induce mitosis but has a unique role in activating cyclin B1-Cdk1 at the centrosome. J. Cell Biol..

[73] Zydek M., Hagemeier C., Wiebusch L. (2010). Cyclin-dependent kinase activity controls the onset of the HCMV lytic cycle. PLoS Pathog..

[74] Kim S., Seo D., Kim D., Hong Y., Chang H., Baek D., Kim V.N., Lee S., Ahn K. (2015). Temporal Landscape of MicroRNA-Mediated Host-Virus Crosstalk during Productive Human Cytomegalovirus Infection. Cell Host Microbe.

[75] Guo X., Huang Y., Qi Y., Liu Z., Ma Y., Shao Y., Jiang S., Sun Z., Ruan Q. (2015). Human cytomegalovirus miR-UL36-5p inhibits apoptosis via downregulation of adenine nucleotide translocator 3 in cultured cells. Arch. Virol..

[76] Wang Y.P., Qi Y., Huang Y.J., Qi M.L., Ma Y.P., He R., Ji Y.H., Sun Z.R., Ruan Q. (2013). Identification of immediate early gene X-1 as a cellular target gene of hcmv-mir-UL148D. Int. J. Mol. Med..

[77] Hancock M.H., Crawford L.B., Perez W., Struthers H.M., Mitchell J., Caposio P. (2021). Human Cytomegalovirus UL7, miR-US5-1, and miR-UL112-3p Inactivation of FOXO3a Protects CD34(+) Hematopoietic Progenitor Cells from Apoptosis. mSphere.

[78] Tothova Z., Gilliland D.G. (2007). FoxO transcription factors and stem cell homeostasis: Insights from the hematopoietic system. Cell Stem Cell.

[79] Liang R., Ghaffari S. (2018). Stem Cells Seen Through the FOXO Lens: An Evolving Paradigm. Curr. Top. Dev. Biol..

[80] Dijkers P.F., Medema R.H., Lammers J.W., Koenderman L., Coffer P.J. (2000). Expression of the pro-apoptotic Bcl-2 family member Bim is regulated by the forkhead transcription factor FKHR-L1. Curr. Biol..

[81] Brunet A., Bonni A., Zigmond M.J., Lin M.Z., Juo P., Hu L.S., Anderson M.J., Arden K.C., Blenis J., Greenberg M.E. (1999). Akt promotes cell survival by phosphorylating and inhibiting a Forkhead transcription factor. Cell.

[82] Tzivion G., Dobson M., Ramakrishnan G. (2011). FoxO transcription factors; Regulation by AKT and 14-3-3 proteins. Biochim. Biophys. Acta.

[83] Yang J.Y., Zong C.S., Xia W., Yamaguchi H., Ding Q., Xie X., Lang J.Y., Lai C.C., Chang C.J., Huang W.C. (2008). ERK promotes tumorigenesis by inhibiting FOXO3a via MDM2-mediated degradation. Nat. Cell Biol..

[84] O’Connor C.M., Vanicek J., Murphy E.A. (2014). Host microRNA regulation of human cytomegalovirus immediate early protein translation promotes viral latency. J. Virol..

[85] Thiel G., Cibelli G. (2002). Regulation of life and death by the zinc finger transcription factor Egr-1. J. Cell Physiol..

[86] Min I.M., Pietramaggiori G., Kim F.S., Passegue E., Stevenson K.E., Wagers A.J. (2008). The transcription factor EGR1 controls both the proliferation and localization of hematopoietic stem cells. Cell Stem Cell.

[87] Krishnaraju K., Hoffman B., Liebermann D.A. (2001). Early growth response gene 1 stimulates development of hematopoietic progenitor cells along the macrophage lineage at the expense of the granulocyte and erythroid lineages. Blood.

[88] Diggins N.L., Crawford L.B., Hancock M.H., Mitchell J., Nelson J.A. (2020). A Novel miRNA-Mediated Mechanism to Retain Latent HCMV Genomes in Hematopoietic Progenitor Cells. Cell Rep..

[89] Grey F., Tirabassi R., Meyers H., Wu G., McWeeney S., Hook L., Nelson J.A. (2010). A viral microRNA down-regulates multiple cell cycle genes through mRNA 5’UTRs. PLoS Pathog..

[90] Fries B.C., Khaira D., Pepe M.S., Torok-Storb B. (1993). Declining lymphocyte counts following cytomegalovirus (CMV) infection are associated with fatal CMV disease in bone marrow transplant patients. Exp. Hematol..

[91] Rakusan T.A., Juneja H.S., Fleischmann W.R. (1989). Inhibition of hemopoietic colony formation by human cytomegalovirus in vitro. J. Infect. Dis..

[92] Maciejewski J.P., Bruening E.E., Donahue R.E., Mocarski E.S., Young N.S., St Jeor S.C. (1992). Infection of hematopoietic progenitor cells by human cytomegalovirus. Blood.

[93] Goodrum F., Jordan C.T., Terhune S.S., High K., Shenk T. (2004). Differential outcomes of human cytomegalovirus infection in primitive hematopoietic cell subpopulations. Blood.

[94] Sing G.K., Ruscetti F.W. (1990). Preferential suppression of myelopoiesis in normal human bone marrow cells after in vitro challenge with human cytomegalovirus. Blood.

[95] Mason G.M., Poole E., Sissons J.G., Wills M.R., Sinclair J.H. (2012). Human cytomegalovirus latency alters the cellular secretome, inducing cluster of differentiation (CD)4+ T-cell migration and suppression of effector function. Proc. Natl. Acad. Sci. USA.

[96] Swirnoff A.H., Apel E.D., Svaren J., Sevetson B.R., Zimonjic D.B., Popescu N.C., Milbrandt J. (1998). Nab1, a corepressor of NGFI-A (Egr-1), contains an active transcriptional repression domain. Mol. Cell Biol..

[97] Thiel G., Kaufmann K., Magin A., Lietz M., Bach K., Cramer M. (2000). The human transcriptional repressor protein NAB1: Expression and biological activity. Biochim. Biophys. Acta.

[98] Buehler J., Zeltzer S., Reitsma J., Petrucelli A., Umashankar M., Rak M., Zagallo P., Schroeder J., Terhune S., Goodrum F. (2016). Opposing Regulation of the EGF Receptor: A Molecular Switch Controlling Cytomegalovirus Latency and Replication. PLoS Pathog..

[99] Hancock M.H., Mitchell J., Goodrum F.D., Nelson J.A. (2020). Human Cytomegalovirus miR-US5-2 Downregulation of GAB1 Regulates Cellular Proliferation and UL138 Expression through Modulation of Epidermal Growth Factor Receptor Signaling Pathways. mSphere.

[100] Hancock M.H., Hook L.M., Mitchell J., Nelson J.A. (2017). Human Cytomegalovirus MicroRNAs miR-US5-1 and miR-UL112-3p Block Proinflammatory Cytokine Production in Response to NF-kappaB-Activating Factors through Direct Downregulation of IKKalpha and IKKbeta. mBio.

[101] Kim Y., Lee S., Kim S., Kim D., Ahn J.H., Ahn K. (2012). Human cytomegalovirus clinical strain-specific microRNAn miR-UL148D targets the human chemokine RANTES during infection. PLoS Pathog..

[102] Hook L.M., Grey F., Grabski R., Tirabassi R., Doyle T., Hancock M., Landais I., Jeng S., McWeeney S., Britt W. (2014). Cytomegalovirus miRNAs target secretory pathway genes to facilitate formation of the virion assembly compartment and reduce cytokine secretion. Cell Host Microbe.

[103] Stern-Ginossar N., Saleh N., Goldberg M.D., Prichard M., Wolf D.G., Mandelboim O. (2009). Analysis of human cytomegalovirus-encoded microRNA activity during infection. J. Virol..

[104] Poole E., McGregor Dallas S.R., Colston J., Joseph R.S.V., Sinclair J. (2011). Virally induced changes in cellular microRNAs maintain latency of human cytomegalovirus in CD34(+) progenitors. J. Gen. Virol..

[105] Nachmani D., Lankry D., Wolf D.G., Mandelboim O. (2010). The human cytomegalovirus microRNAn miR-UL112 acts synergistically with a cellular microRNA to escape immune elimination. Nat. Immunol..

[106] Lee S., Song J., Kim S., Kim J., Hong Y., Kim Y., Kim D., Baek D., Ahn K. (2013). Selective degradation of host MicroRNAs by an intergenic HCMV noncoding RNA accelerates virus production. Cell Host Microbe.

[107] Yang Y., Wang S., Miao Z., Ma W., Zhang Y., Su L., Hu M., Zou J., Yin Y., Luo J. (2015). miR-17 promotes expansion and adhesion of human cord blood CD34(+) cells in vitro. Stem Cell Res. Ther..

